# Population genomics of a natural *Cannabis sativa* L. collection from Iran identifies novel genetic loci for flowering time, morphology, sex and chemotyping

**DOI:** 10.1186/s12870-025-06045-4

**Published:** 2025-01-21

**Authors:** Mahboubeh Mostafaei Dehnavi, Annabelle Damerum, Sadegh Taheri, Ali Ebadi, Shadab Panahi, George Hodgin, Brian Brandley, Seyed Alireza Salami, Gail Taylor

**Affiliations:** 1 Department of Plant Sciences, University of California, Davis, CA USA; 2 Department of Horticultural Science, Faculty of Agriculture, University of Tehran, Karaj, Iran; 3 Department of Animal Science, Faculty of Agriculture, Ferdowsi University of Mashhad, Mashhad, Iran; 4 Biopharmaceutical Research Company, Castroville, CA USA; 5 Industrial and Medical Cannabis Research Institute (IMCRI), Tehran, Iran; 6 Present address, Zymo Research Corp, Irvine, CA USA

**Keywords:** Genotyping-by-Sequencing, GWAS, Cannabis Breeding, Genetic Diversity, Population Structure, Candidate Gene, Phenotype, Sex

## Abstract

**Background:**

Future breeding and selection of *Cannabis sativa* L. for both drug production and industrial purposes require a source of germplasm with wide genetic variation, such as that found in wild relatives and progenitors of highly cultivated plants. Limited directional selection and breeding have occurred in this crop, especially informed by molecular markers.

**Results:**

This study investigated the population genomics of a natural cannabis collection comprising male and female individuals from various climatic zones in Iran. Using Genotyping-By-Sequencing (GBS), we sequenced 228 individuals from 35 populations. The data obtained enabled an association analysis, linking genotypes with key phenotypes such as inflorescence characteristics, flowering time, plant morphology, tetrahydrocannabinol (THC) and cannabidiol (CBD) content, and sex. We detected approximately 23,266 significant high-quality Single Nucleotide Polymorphisms (SNPs), establishing associations between markers and traits. The population structure analysis revealed that Iranian cannabis plants fall into five distinct groups. Additionally, a comparison with global data suggested that the Iranian populations is distinctive and generally closer to marijuana than to hemp, with some populations showing a closer affinity to hemp. The GWAS identified novel genetic loci associated with sex, yield, and chemotype traits in cannabis, which had not been previously reported.

**Conclusion:**

The study's findings highlight the distinct genetic structure of Iranian Cannabis populations. The identification of novel genetic loci associated with important traits suggests potential targets for future breeding programs. This research underscores the value of the Iranian cannabis germplasm as a resource for breeding and selection efforts aimed at improving Cannabis for various uses.

**Supplementary Information:**

The online version contains supplementary material available at 10.1186/s12870-025-06045-4.

## Background

*Cannabis sativa* L. (cannabis) from the Cannabinaceae family can be used as a source of both pharmacologic drugs for the treatment of tumors, schizophrenia and other medical conditions, but also as fiber and oil, depending on the quantities of tetrahydrocannabinol (THC) and cannabidiol (CBD) within any particular plant, landrace or cultivar [1, 2]. *C. sativa* has a long history of cultivation, and it has been suggested that the global cannabis market may be valued annually at over $300 billion in coming years, as many US states and global nations de-regulate the use of this plant-based chemical for pharmaceutical and recreational use [3–5]. Much remains to be discovered concerning the diversity of genetic and chemical signatures across the species of *C. sativa,* and it seems likely that wild populations of previously uncharacterized *C. sativa* can provide a valuable source of natural genetic variation as foundational resources for future directed breeding programs [5, 6]. The plant is an annual species, primarily dioecious and exhibits high levels of heterozygosity. It has a diploid genome (2n=20) estimated to be 843 Mb for male plants and 818 Mb for female plants. Additionally, the species is believed to possess almost 30,000 genes [7–10].

Depending on type and cannabinoid yield, in particular, THC: CBD ratio, this species can be defined as industrial hemp (a major source of textiles, food, and oilseed) or marijuana (medical cannabis or a recreational drug) [5, 11, 12]. There is significant potential in the development of cannabis and its derivatives, particularly CBD, in the treatment of melanoma, a type of skin cancer and epilepsy-related syndromes such as Lennox-Gastaut syndrome and Dravet syndrome [13, 14].

Cannabis breeding to date has been mostly outside of the public domain; therefore, the true genetic diversity of commercial varieties is unknown [8]. Unraveling the genetic information of natural cannabis populations facilitates breeding programs for different industrial and medical purposes [5]. Genotyping by sequencing (GBS) is a highly multiplexed and high-throughput method for determining the genetic structure of an individual or a population [15, 16]. This technique has played a significant role in the advancement of our understanding of the genetic diversity, evolution and breeding of cannabis [17]. Cannabis, as a complex plant species, has a high degree of genetic diversity, which has made it an ideal candidate for GBS studies. One of the main goals of GBS studies in cannabis is to understand links between phenotype and genotype and identify the genetic markers associated with important and complex traits [18].

By phenotyping a large number of plants, researchers can identify the genetic variations that are associated with specific traits and ultimately help to improve the breeding process. The phenotyping of traits such as sex, flowering time, cannabinoids production, flower structure, agronomic-related and disease and pest resistance is crucial for the cannabis industry [19–21]. Accurately identifying and characterizing these traits can inform breeding programs for the development of high-quality cannabis varieties with desirable traits. Early sex determination and the identification of molecular markers associated with sex are critical tools for cannabis growers and breeders looking to produce high-quality and high-yielding crops [22, 23]. Furthermore, understanding the flowering time of a plant is a significant characteristic for not only optimizing yield and determining harvest time, but also the fiber quality and cannabinoids produced [24]. Flower structure and plant height can also affect the cultivation process and crop yield and cannabinoid production, such as THC and CBD, is a key factor in the medicinal and recreational use of cannabis [5, 25, 26]. Therefore, the precise phenotyping of these traits can improve crop management, increase yield and enhance the overall quality of cannabis [27].

Another important application of GBS in cannabis is in the identification and characterization of landrace cultivars. These cultivars have unique genetic profiles and are important sources of diversity for breeding programs [5]. GBS studies have helped identify the genetic relationships between landraces and characterize their genetic diversity [28].

Genetic investigation of different natural resources assists in the development of pre-breeding and the identification of new varieties for research purposes and enables the initiation of a pipeline of novel discovery toward commercialization [29, 30]. In this study, we conducted genotyping of both male and female cannabis genomes to enhance our comprehension of cannabis sex evolution, as well as cannabinoid expression. We used GBS to characterize natural cannabis plant material obtained from various regions of Iran. We employed a set of 23,266 significant SNP markers, which were linked to various essential features, such as THC and CBD content (which distinguishes the drug from the hemp chemotype), sex expression, flowering time, female inflorescence features and some other morpho-physiological traits such as plant height, number of nodes, number of leaves, internode length and footstalk diameter. The investigation offers insight into population structure, genetic relationship, and genetic diversity of the cannabis species.

## Results

### Phenotyping, genotyping and data quality control

**Fig. 1:**
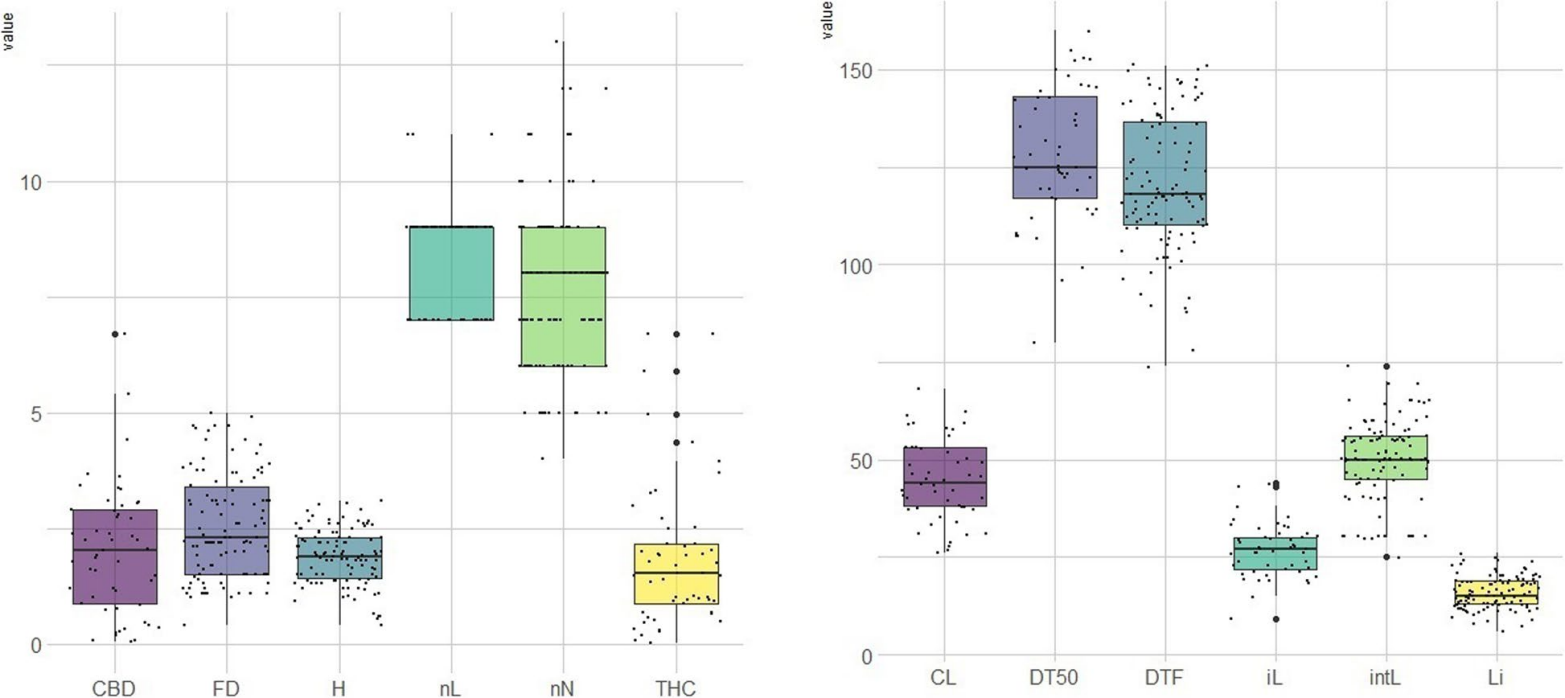
Box plot for investigated traits of cannabis populations used in this study. Cannabidiol quantity (CBD), footstalk diameter (FD), plant height (H), number of leaves (nL), number of nodes (nN), ∆9- tetrahydrocannabinol quantity (THC), crown length (CL), number of days to 50% flowering (DT50), number of days to the initiation of flowering (DTF), inflorescence length (iL), internode length (intL), number of lateral pistillate inflorescences (Li). The diagrams were generated using R (V4.3.1)

Box plots illustrate the phenotypic variation for all the traits investigated (Fig. 1). Most traits exhibited a normal distribution, with the exceptions of DT50, THC Q, CBD Q, nL, and DTF (Fig. S1). Sex was not included as it is not feasible due to its non-quantitative nature. We observed great phenotypic variation within our cannabis populations. The footstalk diameter exhibited substantial diversity, ranging from 1.62 to 3.66, with an average of 2.54, showing the highest coefficient of variation at 31.32%. In contrast, THC and CBD concentrations displayed narrower variations, ranging from 1.17 to 3.13% for THC and 0.96 to 6.69% for CBD, with averages of 1.96% and 1.65%, respectively. Consequently, they exhibited the lowest coefficients of variation at 0.51% and 0.52%, respectively (Table 1). The phenotypic data collected for the GWAS panel, along with the SNP-based heritability (h^2^) measurments are summarized in Table 1. The sequencing data of this study is available at https://www.ncbi.nlm.nih.gov/sra/PRJNA1076947. A total of 1.456 billion reads of 100 bp length were obtained from the four sequencing lanes on HiSeq^TM^ 4000. After quality control and trimming for barcode adapter sequences, an average of approximately 4 million reads per sample were retained for the first (3,883,851 reads/sample; 80.6% of original reads), second (3,854,236 reads/sample; 82.8%), and third (3,935,551 reads/sample; 79.5%) libraries, while approximately 10 million reads (10,774,545 reads/sample; 64.3%) remained for the fourth library, which included only 35 pooled samples. The number of reads retained per sample ranged between 108K and 104M reads, with samples with <100K reads removed. The average quality scores, Q30 ratio and guanine–cytosine (GC) content of the reads were ~39, ~96% and 44.1%, respectively. On average, 80.11% of the reads aligned with the *C. sativa* cs10 reference genome [8]. Differences in sequencing depth across regions, excessive PCR amplification, short read length, or issues with the sequencing platform may have contributed to the variations observed in the percentage of reads mapped to the reference. Specifically, the minimum mapping percentage recorded was 50.09%, which was observed in the case of sample ID 218, representing one of the individuals from population Bsh-01. Following filtration, a total of 23,266 high-quality SNPs were identified across Iranian samples, which were subsequently selected for the analysis of population structure and marker-trait association. Additionally, a set of 25,112 informative SNPs were retained after applying filtration to the combined datasets, which included the previously sequenced Iranian samples and collections of hemp and marijuana.

**Table 1:** Characteristics studied in the cannabis populations (sex trait has not been reported here as it is not a quantitative trait)

**No.**	**Trait**	**Abbr.**	**Unit**	**Min.**	**Max.**	**Mean**	**SD**^**a**^	**CV**^**b**^** (%)**	**h**^**2c**^
1	Inflorescence length	iL	cm	23.78	31.32	27.86	3.84	14.32	0.426
2	Number of lateral inflorescences	Li	count	12.37	19.44	15.79	2.75	17.77	0.389
3	Number of nodes	nN	count	6.27	9.03	7.52	1.06	14.19	0.462
4	Height	H	m	1.27	2.68	1.92	0.49	27.37	0.341
5	Number of leaves	nL	count	7.57	8.57	8.19	0.54	6.66	0.385
6	Internode length	intL	cm	41.41	59.03	50.43	6.53	13.56	0.314
7	Footstalk diameter	FD	cm	1.62	3.66	2.54	0.73	31.32	0.382
8	Number of days to first blooming	DTF	day	101.55	136.10	121.71	12.87	10.54	0.301
9	Number of days to 50% blooming	DT50	day	117.5	142.35	129.97	11.45	8.96	0.339
10	Crown length	CL	cm	36.28	54.53	44.93	8.55	19.63	0.361
11	∆9-THC Quantity	THC Q	%	1.17	3.13	1.96	0.92	0.51	0.342
12	CBD Quantity	CBD Q	%	0.96	6.69	1.65	0.70	0.52	0.373

^a^SD -(standard deviation)

^b^CV - (coefficient of variation), estimated as the ratio of the standard deviation to the mean of all populations

^c^h^2^- (SNP-based heritability), the proportion of phenotypic variance explained by all measured SNPs

### Population structure analysis

**Fig. 2:**
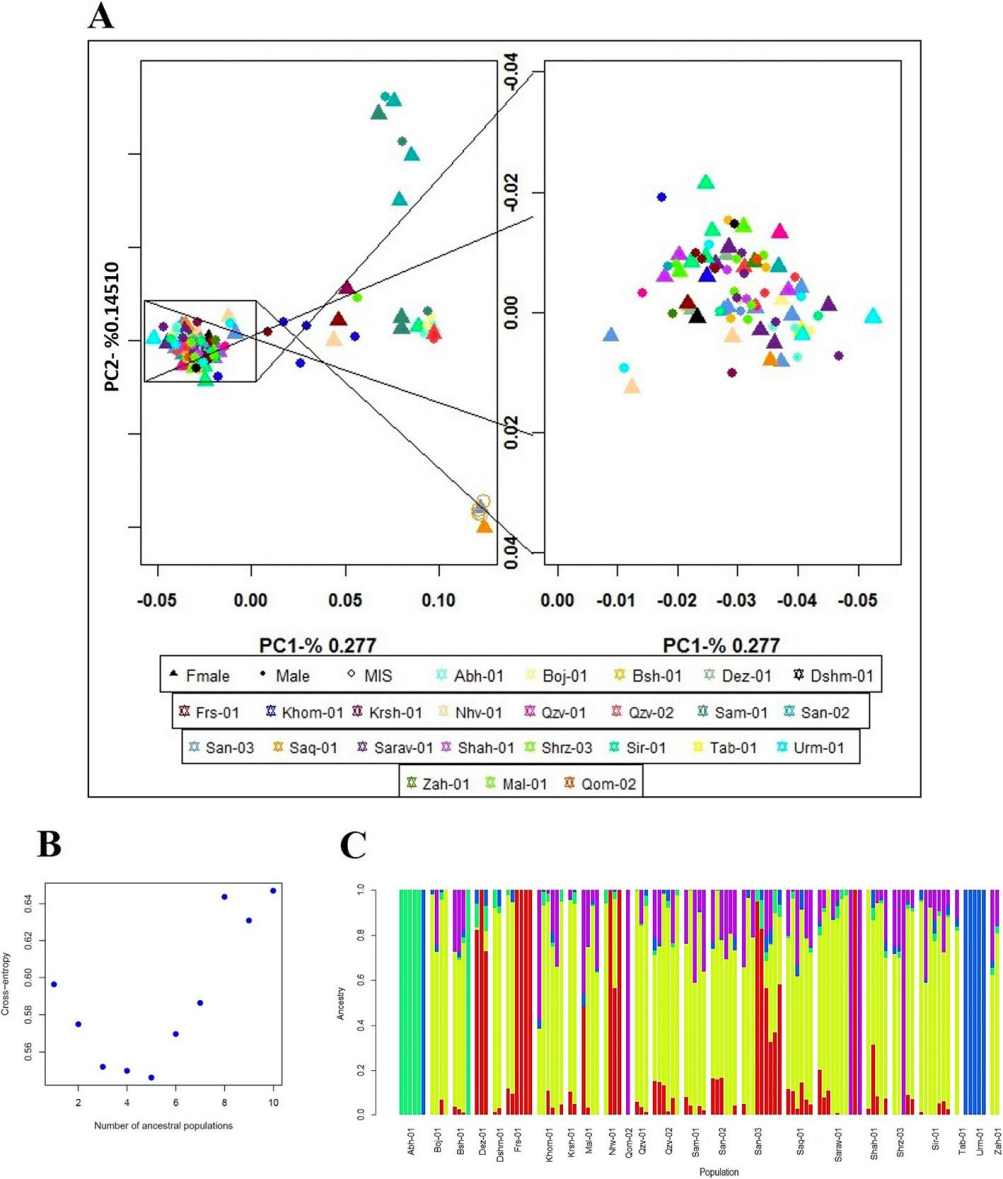
(**A**) Individual-based principal components analysis for Iranian regions using 23,266 SNPs, MIS represents the samples with unknown sex, triangle and circle represent female and male individuals respectively, (**B**) K values plot from K= 1 to K= 10 based on cross-validation error, and (**C**) Q estimates bar plot of studied populations in R (V4.3.1) at K = 5. Each vertical bar exemplifies a genotype

**Fig. 3:**
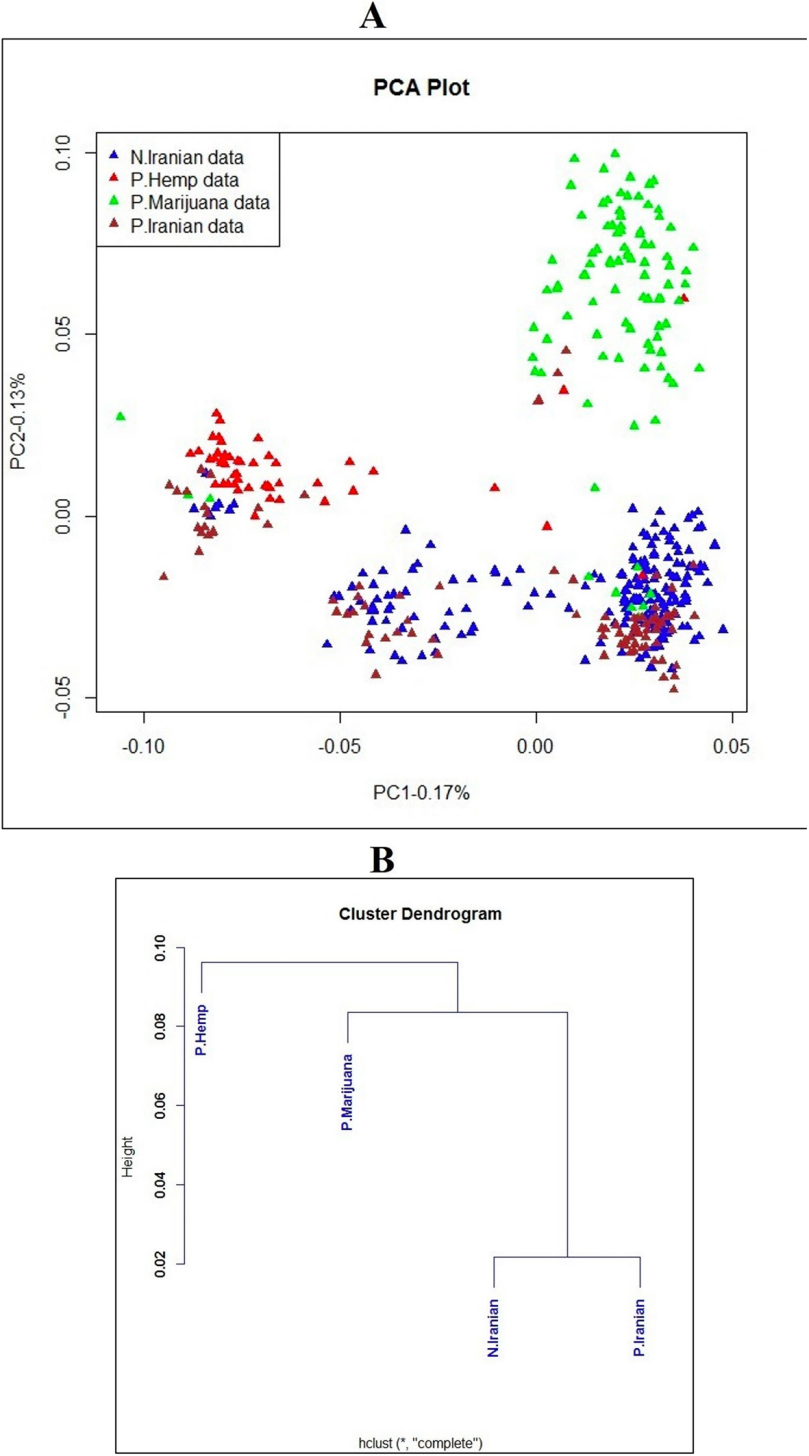
(**A**) Principal components analysis of 431 samples including 196 Iranian samples of this study (in blue), 93 previous studied cannabis samples (in dark red), 47 hemp samples (in red) and 95 marijuana samples (in green) using 25,112 SNPs, and (**B**) Cluster Dendrogram generated for genetic distance between Iranian cannabis genotypes and hemp and marijuana populations. The labels N.Iranian data, P.Iranian data, P.Hemp data and P.Marijuana data represent the following: Iranian samples from the current study, Iranian samples sourced from a prior study, a previously studied hemp population and a previously studied marijuana population respectively

Principal Component Analysis (PCA) findings indicated a high level of genetic similarity among these populations (Fig. 2A). The Sqz-01 population and some individuals of San-02 failed to group with other clusters (Fig. 2A). Admixture's cross-validation procedure was used to determine the most likely number of genetic groups (K). The population structure of studied samples was described by testing the probable number of clusters (K) from 1 to 10, with K= 5 selected as the optimal representation of ancestral populations based on the lowest cross-entropy criterion and visualized using a Q estimates bar plot (Fig. 2B-C). The PCA analysis of integrated data with two public datasets revealed that while the Iranian samples exhibited distinct genetic differences from the hemp and marijuana populations, they showed generally closer genetic proximity to the marijuana population. However, some individual Iranian samples exhibited a closer genetic resemblance to hemp (Fig. 3A). These findings were further supported by the dendrogram plot, which indicated that the genetic distance between the Iranian samples from this study and the previously studied marijuana population was smaller than the distance between the Iranian samples from the current study and the previously studied hemp population (Fig. 3B).

### Genetic variation and differentiation

**Fig. 4:**
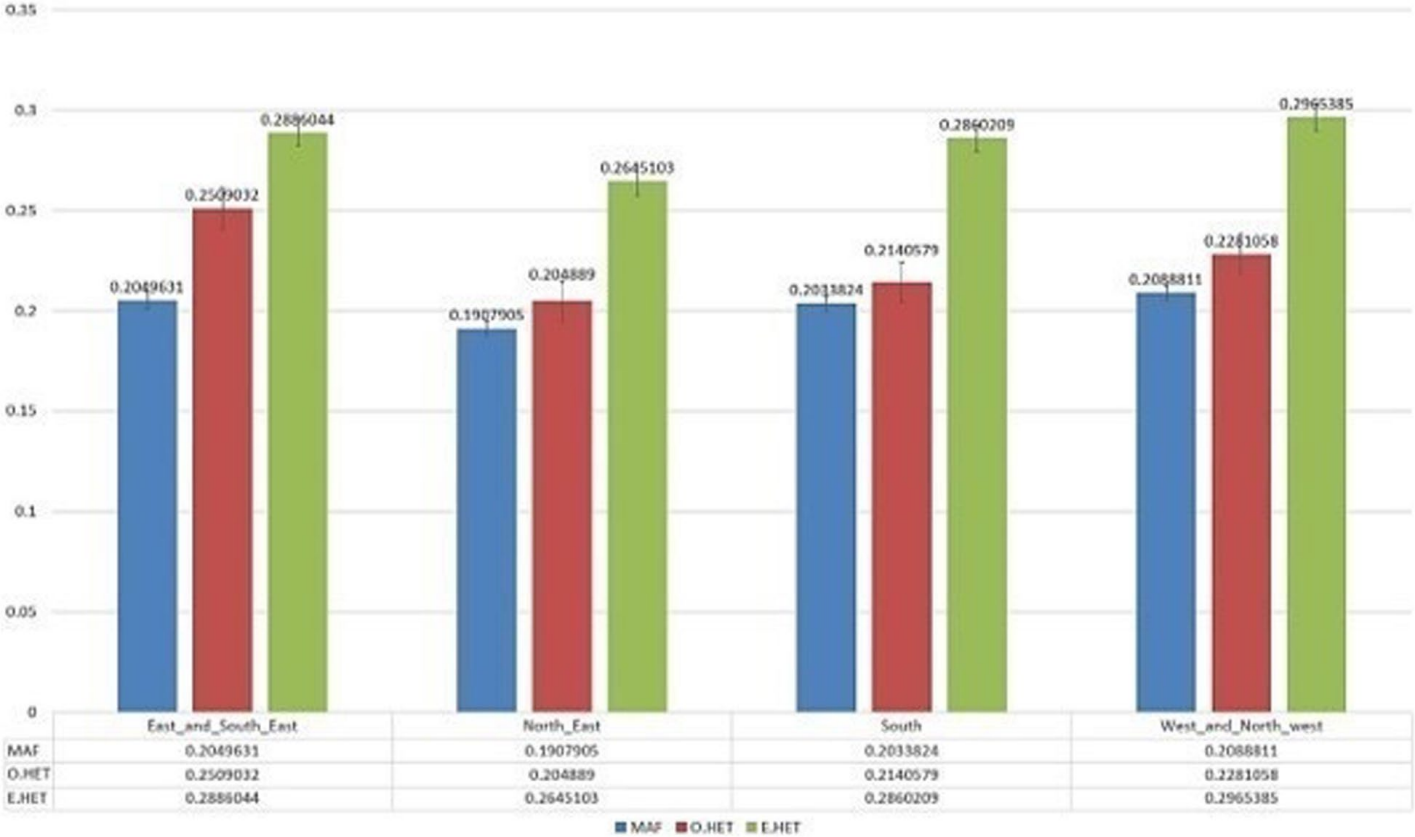
Minor Allele Frequency (MAF), Observed heterozygosity (O.HET) and expected heterozygosity (E.HET) obtained for the populations of east and southeast, northeast, south and west and northwest of Iran

To assess the genetic differentiation among Iranian cannabis populations, we calculated the genetic differentiation parameter (F_ST_) as well as observed and expected heterozygosity for each pairwise comparison. Initially, we generated six F_ST_ plots to compare distinct populations of Iranian samples (Fig. S2). The observed heterozygosity (H_o_) for these groups was found as 0.25 (for the east and southeast population), 0.204 (for the northeast population), 0.214 (for the south population), and 0.228 (for the west and northwest). Corresponding, the respective expected heterozygosity (H_e_) values were obtained as 0.288, 0.264, 0.286, and 0.296, while the estimated minor allele frequency (MAF) were 0.204, 0.19, 0.203, and 0.208. The details of observed heterozygosity, expected heterozygosity, and minor allele frequency are provided in Fig. 4.

Our study exhibited higher heterozygosity than that earlier study on Iranian samples observed by Soorni et al. [28]. Our results showed a similar level of heterozygosity to the hemp accessions studied by Sawler et al. [17], while the earlier Iranian sample study reported heterozygosity more similar to the marijuana accessions studied in the same study.

Figs. S1-S3 present F_ST_ Manhattan plots and correlation plots for all possible pairings of these populations. Significant SNP markers along with their corresponding loci, gene annotations, and the F_ST_ values across all F_ST_ analyses are provided in Tables S2-S18.

The pairwise comparisons among the geography-based Iranian populations in this study revealed that the east and southeast population and northeast population had the highest number of SNP markers (95 SNPs) (Fig. S2C; Table S2), followed by the northeast population and south population with 89 SNP markers which related to 32 specific loci (Fig. S2F; Table S3). On the other hand, the east and southeast population and southern population exhibited the lowest number of SNPs (29 SNPs) that were linked to five specific loci (Fig. S2D; Table S5).

When comparing Iranian populations with global collections, in contrast to the hemp collection, the marijuana collection exhibited a larger number of SNP markers concerning the geography-based Iranian populations. Among these comparisons, the northeast population of Iran demonstrated the highest number of SNP marker (134 SNPs) spanning 36 loci in comparison to the marijuana collection (Fig. S2A; Table S13). Furthermore, the northeast population emerged with the highest number of SNP markers (112 SNPs) linked to 49 loci when compared to the hemp collection (Fig. S2C; Table S9). Overall, the northeast population of Iran appeared to be more distinct not only from other populations within Iran but also from both the hemp and marijuana collections.

In a separate analysis, pairwise comparisons were conducted between the Iranian samples from this study as a single population and previously studied Iranian samples, as well as hemp and marijuana collections. It was found that there were less SNP markers shared between Iranian samples and hemp (107 SNPs) (Fig. S4A; Table S16) than between Iranian samples and the marijuana collection (113 SNPs) (Fig. S4B; Table S17). In both of these comparisons, a total of 37 loci were found to exhibit differences (Fig. S4A and 3B; Tables S16 and S17). Of these, six loci were identified as being common to both comparisons, including LOC115694687, which encodes separase (RefSeq accession: XM_030621771.1), LOC115707169 encoding for histone deacetylase 2 (RefSeq accession: XM_030635038.1), LOC115707184 encoding for spidroin-2 (RefSeq accession: XM_030635066.1), LOC115707237 encoding for serine/threonine protein phosphatase 2A 55 kDa regulatory subunit B (RefSeq accession: XM_030635130.1), LOC115725648 with an uncharacterized description (RefSeq accession: XM_030655228.1), and LOC115725736 encoding for partner of Y14 and mago (RefSeq accession: XM_030655329.1). It is noteworthy that the comparisons unveiled 134 SNP markers associated with 51 loci that exhibited differences between the Iranian samples of this study and the previously studied Iranian samples. These SNPs were distributed across chromosomes 1 (*n*=5), 2 (*n*=11), 4 (*n*=31), 5 (*n*=27), 7 (*n*=6) and 10 (*n*=54) (Fig. S 4C; Table S18). This suggests the existence of genomic differences between these two sample sets.

In the first F_ST_ analysis conducted among the four geographical-based Iranian populations, the highest and lowest F_ST_ values were observed for the east and southeast: northeast pair (F_ST_= 0.09) and northeast: west and northwest pair (F_ST_= 0.024), respectively. A higher F_ST_ value indicates greater genetic differences between populations. Moving on to the second F_ST_ analysis, which incorporated global data, the pairs of marijuana: northeast demonstrated the lowest F_ST_ value (F_ST_= 0.06), while the pair of hemp: east and southeast exhibited the highest F_ST_ value (F_ST_= 0.17). These results suggest that Iranian populations displayed higher F_ST_ values compared to hemp rather than marijuana. Furthermore, in the last set of F_ST_ comparisons, it was found that the F_ST_ estimation between Iranian samples as a single population and the hemp collection (F_ST_= 0.086) was higher compared to the F_ST_ value between Iranian samples and the marijuana population (F_ST_= 0.062). Additionally, the F_ST_ value between the Iranian samples from this study and the Iranian samples from previous studies was 0.015. Overall, these findings indicate that Iranian populations are genetically closer to marijuana than hemp. Moreover, the SNP markers that revealed differences within Iranian populations were predominantly concentrated on Chromosomes 3, 4, and 1, indicating potential genomic regions that contribute to genetic variation and genotype differentiation. These specific chromosomes may harbor genes or regulatory elements that play a significant role in shaping the unique genetic landscape of Iranian populations, warranting further investigation to uncover the underlying genetic mechanisms and potential functional implications of these variations.

### Genotype and phenotype associations

**Fig. 5:**
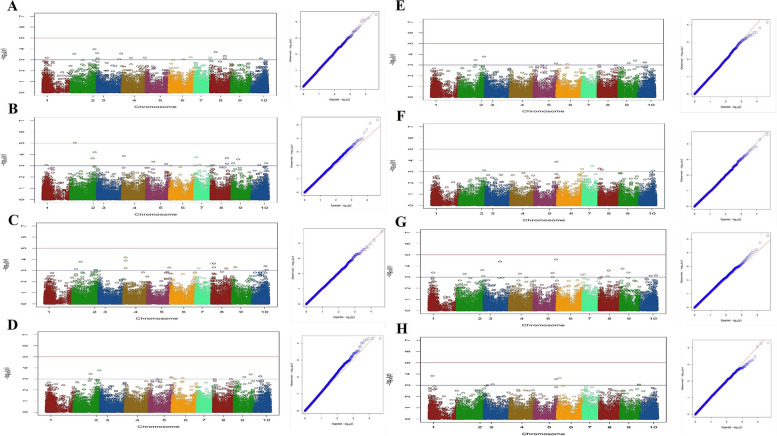
Manhattan plots (left) and quantile–quantile plots (right) of association analysis for traits: (**A**) H (**B**) nN (**C**) Li (**D**) iL (**E**) DTF (**F**) DT50 (**G**) FD and (**H**) nL in studied Iranian cannabis collection

A total of 193 significant SNP associations were detected for the investigated traits. Among these, 47 SNPs were identified within annotated genes. Overall, the study revealed a total of 47 candidate loci related to the traits under investigation, out of which seven genes remain uncharecterized. Detailed information for all identified significant SNPs, is provided in Table S1 and Table 2 with associated candidate loci, and annotation information presented specifically for those SNPs located within annotated genes. Specifically, 18 SNPs were found to be associated with H (Fig. 5A). Among these SNP markers, five markers, two on chromosome 3 (chr3_281101_71, chr3_288389_58), two on chromosome 4 (chr4_327964_19, chr4_382581_26), and one on chromosome 2 (chr2_84187_7) were linked to annotated genes (Table S1). 19 SNP markers were found to be associated with the nN, while 17 SNP markers were associated with the Li. Specifically, for the nN, there were five SNP markers located on chromosomes 2, 4, 5 and 9 that were located within annotated genes (Fig. 5B). (Table S1). Similarly, for the Li, there were two markers on chromosomes 5 and 9 that were identified within annotated genes (Fig. 5C; Table S1). Among 10 markers identified as being associated with the iL, a total of four SNP markers positioned on chromosomes 2, 9 and 10 were found to be related to genes (Fig. 5D; Table S1).

**Table 2:** Functional annotations of the significantly associated SNPs for sex trait in Iranian cannabis collection

**CHR**	**SNP ID**	**Position**	***P*-value**	**MAF**	**Gene ID**	**RefSeq Accession**	**Gene Annotation**
1	786516_36	4175218	0.000969	0.33			
1	786516_49	4175231	0.000969	0.33			
1	786516_53	4175235	0.000969	0.33			
2	78363_79	87241293	0.000352	0.42	LOC115721262	XM_030650525.2	uncharacterized LOC115721262
2	109511_15	101011485	0.000414	0.12			
9	585232_75	31767032	0.00059	0.24	LOC115722331	XM_030651522.2	CRM-domain containing factor CFM3A, chloroplastic/mitochondrial
10	666147_74	42986208	0.000558	0.27			
10	689517_88	55869380	0.000621	0.33			

*CHR* Chromosome, *MAF* Minor allele frequency

Significant associations were observed between flowering time-related traits, including DTF and DT50 (Figs. 5E and F). 16 markers were associated with DTF, while eight markers were found to be associated with DT50 (Figs. 5E and F; Table S1). The SNPs associated with DTF were found to be linked with candidate loci, including glutamate--glyoxylate aminotransferase 2, YTH domain-containing protein ECT4, ribonuclease H2 subunit B, and one uncharacterized gene. As for the DT50 trait, only one marker located on chromosome 5, encoding the putative pentatricopeptide repeat-containing protein At2g02150, was identified (Table S1).

Among 17 SNP markers that were detected to be associated with the FD, six SNPs, two on chromosome 5 and one each on chromosomes 2, 3, 4, and 10 were linked to annotated genes (Fig. 5G; Table S1). Also, four SNP markers on chromosomes 3 (*n*=2), 5 (*n*=1) and 6 (*n*=1) from a total of seven markers were detected to be associated with annotated genes for the nL trait (Fig. 5H; Table S1).

**Fig. 6:**
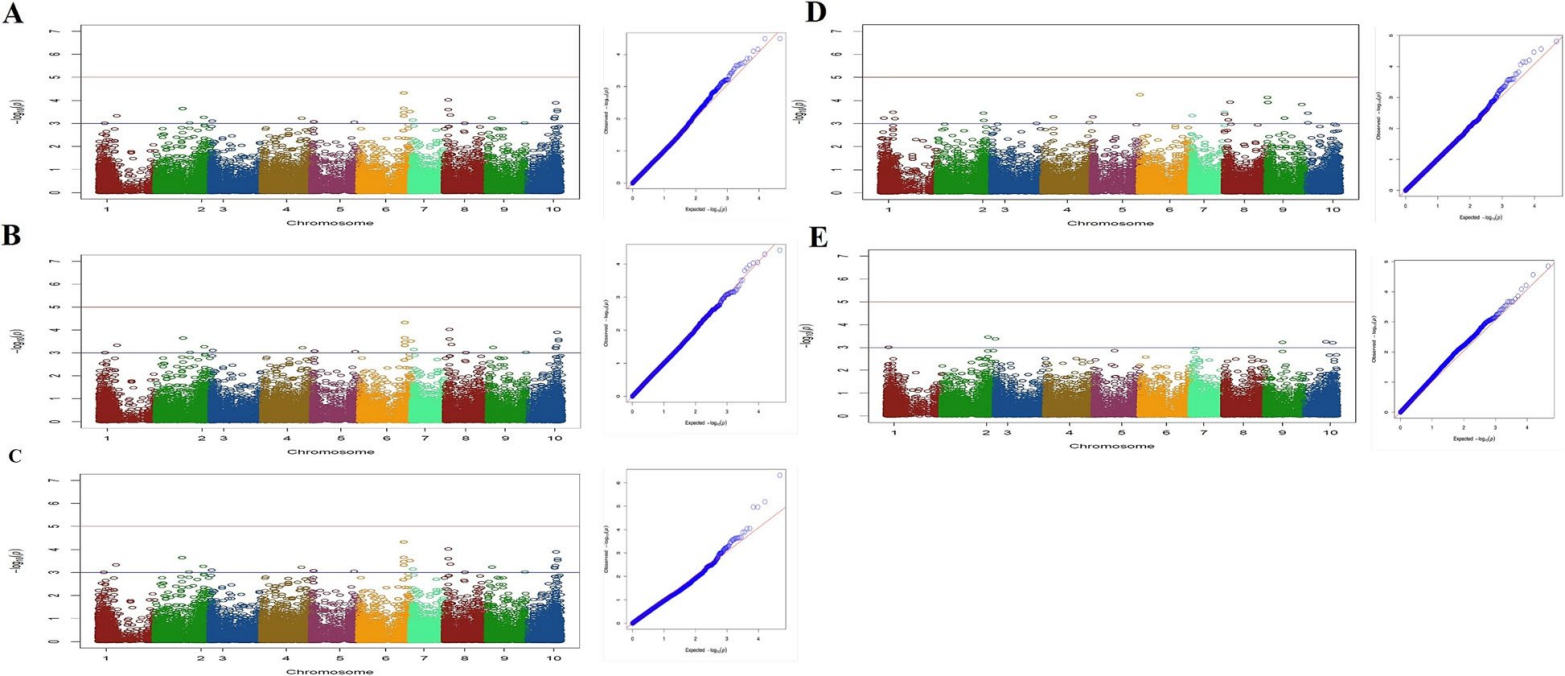
Manhattan plots (left) and quantile–quantile plots (right) of association analysis for traits: (**A**) intL (**B**) CL (**C**) THC Q (**D**) CBD Q and (**E**) F&M in studied Iranian cannabis collection

For the intL trait, seven markers positioned on chromosomes 3, 4 and 5 were associated with genes (Fig. 6A; Table S1). These markers were part of a total of 35 markers identified to be linked with this trait. Regarding the CL trait, among 12 markers that were found to be associated with it, five markers- three on chromosome 3 and two on chromosome 4 were related to annotated genes (Fig. 6B; Table S1).

Eight markers, two on chromosome 1, two on chromosome 3, two on chromosome 8 and one each on chromosomes 7 and 10 were detected to be associated with THC Q trait (Fig. 6C; Table S1). Furthermore, in the case of CBD Q, a total of 24 SNPs were identified. These markers were distributed across whole genome (Fig. 6D). The candidate loci identified to be associated with CBD Q trait included the berberine bridge enzyme-like D-2 (chromosome 7), 3-ketoacyl-CoA synthase 19 (chromosome 1), serine/threonine-protein kinase AtPK1/AtPK6 both (chromosome 3), fimbrin-2 (chromosome 4), endonuclease MutS2 (chromosome 5), small nucleolar RNA R71 (chromosome 2), transcription factor MYB14 (chromosome 9), and one uncharacterized locus (chromosome 4) (Table S1).

It appears that THC and CBD concentrations have complex genetic architectures that extend beyond the already identified cannabinoid synthase genes and are distributed across various chromosomes of the whole genome.

The SNPs associated with sex determination were primarily distributed on the sex chromosome (cs10 v.1.0 chromosome 1 and cs10 v.2.0 chromosome 10), previously identified by Prentout et al. [23], Kovalchuk et al. [30], and McKernan [19], but additional SNPs were also identified at different positions. Eight SNP markers discovered for this trait, which are located on chromosomes 1 (*n*=3), 2 (*n*=2), 9 (*n*=1) and 10 (*n*=2). Among these, two SNPs located across chromosomes 2 and 9 were found to be specifically linked to candidate genes associated with sex (Table 2; Fig. 6E). The Manhattan plots, along with the corresponding quantile-quantile (Q-Q) plots for each trait, were presented in Figs. 5A-H and 6A-E.

## Discussion

This study adds significantly to our limited understanding of the population genomics of *Cannabis sativa* and provides novel insights into gene-trait associations for a natural collection originating from contrasting climatic zones across Iran. The scarcity of such studies likely results from the historical constraints in accessing wide cannabis populations that capture natural genetic diversity [5, 8, 31, 32]. These insights are new, with few previously published GWAS and population genomics studies available for this largely undomesticated crop, and they add important knowledge to our developing understanding of the genomics of medicinal and industrial characterization of the crop. They will help underpin directed breeding programs to enhance traits of interest for commercial production [8] and also add new insight to the population genomics and domestication history recently reported for *C. sativa* [12] that did not include data from the native populations of Iran, such as those reported and characterized here. In a previous study, an assessment of phenotypic diversity in some indigenous cannabis populations in Iran was conducted, identifying key traits and their heritability, which can inform breeding programs for developing new cultivars [33]. However, their work lacks an exploration into the genetic foundations of these traits to create molecular markers and speed up breeding efforts. In contrast, our study has not only obtained genomic and phenotype data but also established a research platform that links genomic variation with germplasm collection, facilitating selections for molecular breeding.

The investigation of genetic variation between domesticated and natural populations is crucial for comprehending the patterns of local adaptation and identifying the genetic sources of desirable characteristics [5]. Studies on wild landraces are emerging, for example, for the wild population of Cannabis in China [34], which revealed five distinct groups and the population genomics for this important site of Cannabis domestication is important, since China is believed may possibly be one of the main centers of origin for this crop [35, 36].

Genotyping of this Iranian natural collection using GBS allowed us to conduct genetic diversity (genetic distance), population structure and genome-wide association analysis among these native populations, such as has been performed in wild (feral) cannabis collections as well as in other species, where wild progenitor populations have been used to inform breeding [11, 37–44]. The results of population structure revealed the presence of five clusters, in contrast to the two genetic clusters reported in the previous investigation of Iranian cannabis populations conducted by Soorni et al. [28]. The estimation of K is influenced by two crucial factors: the number of populations and the genetic dispersion among them [45]. The observed variation may be attributed to the inclusion of a broader range of populations situated in diverse climatic zones and a larger sample size per population. Additionally, the Sqz-01 population did not form a cluster with other population groups. As previously stated, this population stands out due to distinctive morphophysiological characteristics, such as dwarf stature, early flowering, and compact inflorescence, which differentiate it from the other populations [5].

Due to its predominantly wind-pollinated dioecious nature, cannabis is a highly heterozygous and outcrossing species [46, 47]. Sawler et al. [17] noted greater heterozygosity in hemp than in marijuana. Heterozygosity across distinct geographically-based Iranian populations showed a similarity in the heterozygosity to that of hemp accessions studied by Sawler et al. [17], while the earlier study on Iranian samples reported an average heterozygosity which is more similar to that observed in marijuana. This is despite the fact that Lynch et al. [46] observed a significant rise in heterozygosity within drug-type varieties compared to hemp varieties. These distinctions highlight the complex interplay between genetic backgrounds and environmental factors, resulting in diverse heterozygosity patterns within cannabis populations [35]. The expected heterozygosity values were higher than the observed heterozygosity in all four geography-based groups of Iranian populations, possibly indicating the impact of inbreeding and reduced genetic variability [48]. However, the limited sample size of our cannabis collection may also have contributed to these results and increasing the sample size of the population could further improve the results [49–52].

Lower F_ST_ values between Iranian population pairs ranging from 0.02 to 0.06 indicate no strong genetic differentiation among these populations. This phenomenon could be attributed to the distribution of pollen and seeds and the gene flow between these areas [53]. These seed exchanges could also have been facilitated by human activity, particularly for distant locations, as well as by wind pollination and seed dispersal by bird movements [54]. In the earlier investigation by Soorni et al. [28], it was similarly noted that the anticipated lack of significant population differentiation is a result of the wind-pollination characteristic of all known cannabis cultivars, coupled with their notable heterozygosity. To ensure the preservation of their genetic uniformity, numerous marijuana strains are propagated clonally rather than through seed.

GWAS has previously been successful in identifying genotype–phenotype associations in hop (*Humulus lupulus*) and cannabis [55]. Moreover, one of the earliest studies of the cannabis genome and transcriptome found that the genes responsible for producing THC and CBD were located in different regions of the genome than previously thought and that there have been extensive rearrangements and variations in these regions among different cultivars of the plant [56]. For marker–trait association in our study, multiple proteins across the genome are involved in traits related to cannabinoid yields, such as THC and CBD content. This implies that the production of cannabinoids is not only directly tied to the genes responsible for their synthesis in the cannabinoid pathway [8, 17], but rather that additional areas of the genome also control these biosynthetic pathways [47, 57, 58]. This finding aligns with the earlier studies, further supporting the notion that multiple genetic factors contribute to cannabinoid content variation [3, 59–63]. Here we contribute further to this literature by confirming some of these important loci, but also identifying other novel loci for targeted future selection and breeding.

Furthermore, an additional SNP marker −816770_13- located on chromosome 1 and associated with the locus LOC115705717 (RefSeq accession: XM_030633131.1), responsible for 3-ketoacyl-CoA synthesis 19, has been found to be linked with CBD concentration. This SNP was previously identified as being connected to Autoflower1, which is involved in regulating the flowering time of hemp [24]. These findings suggest that certain genes involved in the regulation of flowering time may also be correlated with cannabinoid content. One limitation of this study is that the phenotypic data collected here are only reported for a single growing season and environment, and thus, some caution must be used in generalizing the importance of these findings. However, given that our study confirms earlier identified genetic loci, such as those for flowering linked to cannabinoid production, this gives us confidence that our approach, is worthy and valid but should, in the future, be complemented with additional field trials and analyses to confirm these and other genetic loci.

Regarding the sex trait, the significant SNPs were annotated to uncover potential genetic mechanisms associated with sex determination. While a total of eight potential SNPs located on different chromosomes were identified for this trait, the prior study by Soorni et al. [28], which failed to pinpoint distinct alleles for the regions responsible for sex determination. As the results of this study and earlier studies showed, the process of sex determination in cannabis is intricate and is not solely related to sex chromosomes [19]. It appears that the sex determination mechanism may be influenced by environmental factors and chemical applications [64, 65] and involves the participation of other candidate genes like those related to trichome growth, sex determination, hermaphroditism, and photoperiod independence [8] or genes involved in regulating phytohormone balance and the development of male flowers in female plants [21]. For example, the gene LOC115720754 (RefSeq accession: XM_030649931.1), located on chromosome 2, encodes the Zinc finger protein GAI-ASSOCIATED FACTOR 1, which acts as a transcription factor and a positive regulator of gibberellin (GA) action, homeostasis, and signaling in Arabidopsis [66]. This candidate gene was found to be associated with flowering time [67]. In plants, GA has a role in flowering control [68], and Petit et al. [21] linked the control of the flowering pathways in cannabis to that of sex determination, further highlighting the complexity of these inter-linked traits, where further research is warranted.

Out of the eight candidate loci identified for plant height, we observed that LOC115709353 (RefSeq accession: XM_030637438.1), located on chromosome 3, encodes anaphase-promoting complex subunit 8. In Arabidopsis, this protein is known to play a role in various aspects of development and embryogenesis by regulating the cell cycle, cell division, cell elongation, and endoreduplication control [69–71]. While QTL analysis has been previously conducted for a range of agronomic traits on a population of 375 individuals [27], it has not been performed for the unique array of morpho-physiological traits presented here. These triats include, the number of nodes, internode length, crown length, and the number of leaves, as well as inflorescence- related features such as the number of lateral inflorescences and inflorescences length. The identified SNP markers for these traits have not been previously mapped, and consequently, this study stands as the inaugural attempt to assess these specific traits through association analysis, with loci and candidate genes reported, using the recently available genome sequence of *C. sativa*. The significant SNPs identified are novel for a range of traits and are not shared among them. These SNPs have the potential to serve as markers for marker-assisted breeding in cannabis, pending proper validation.

## Conclusion

Using GBS data from a diverse Iranian cannabis collection of wild germplasm (CGRC), this study has provided significant insights into the genetic variation and differentiation, population structure, and genotype-phenotype associations for this novel germplasm and how it differs from currently available global hemp and marijuana collections. Population structure analysis revealed five distinct groups in the Iranian cannabis collection. Pairwise F_ST_ comparisons identified the northeast population of Iran as the most genetically distinct, making it a priority for future breeding programs. Furthermore, the study confirmed several gene targets for unique traits, including inflorescence features, flowering time, cannabinoid content, sex, and some morphological traits. Together, this study has created a research platform that can link genomic variation and germplasm collection, facilitating selections for molecular breeding. These findings have important implications for improving the quality and productivity of new commercial cannabis varieties through breeding.

## Methods

### Plant material and field experiment

**Fig. 7:**
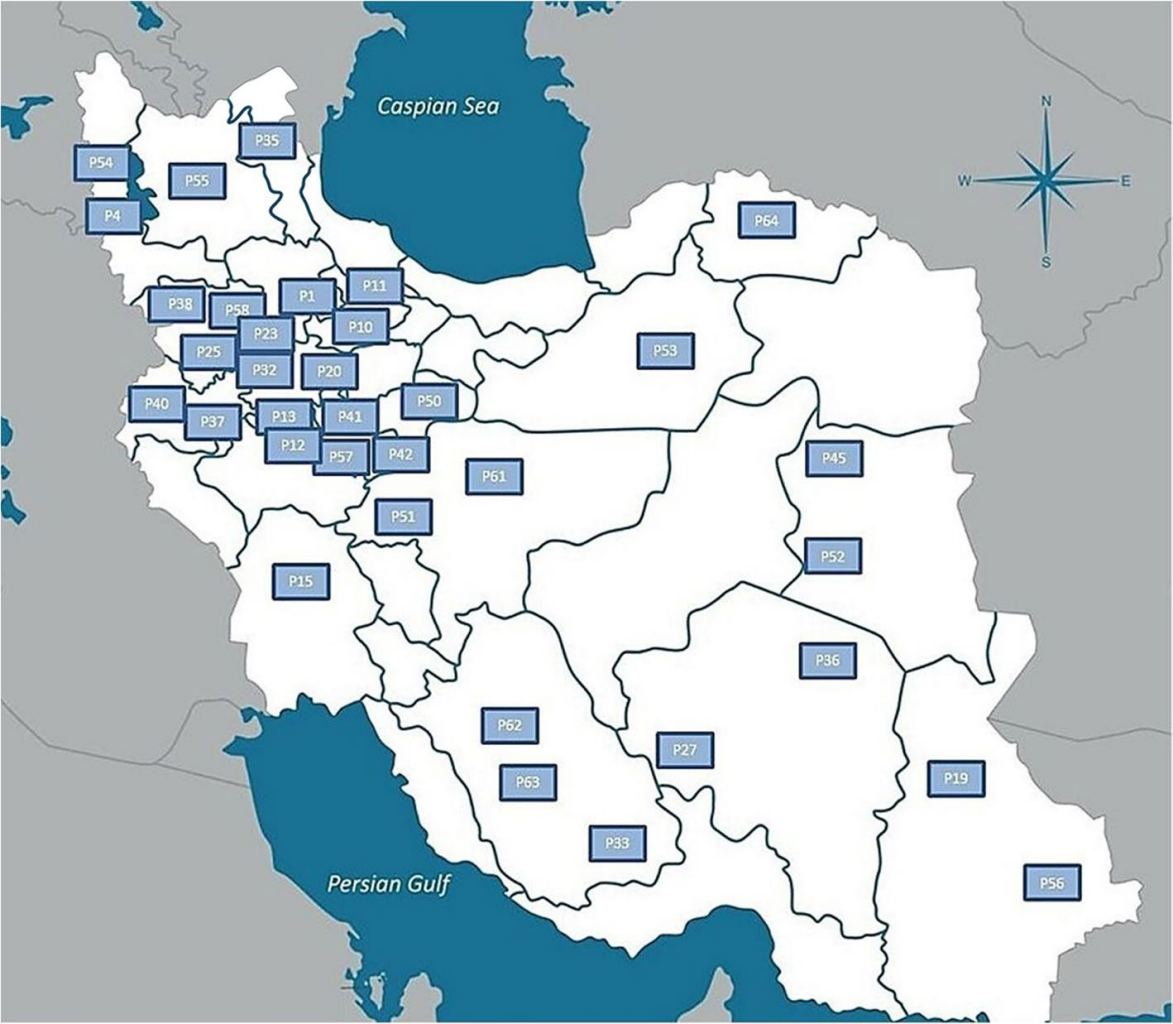
Collection sites and geographical distribution of the studied cannabis populations across regions of Iran. The labels on the map represents the code of each population

Seeds from 35 natural cannabis populations sourced from various locations in Iran, obtained from CGRC (www.medcannabase.org), were grown in the research field of the University of Tehran. The cultivation followed a randomized complete block design with three replicates per population. The separation between each block was set at 2.5 m. Within each block, three rows were arranged for each plot (population), each extending 10 m in length, and with a row spacing of 60 cm and plant spacing of 90 cm, with a total of 10 plants planted across each row. A drip irrigation system was implemented, and plants were grown in soil amended with compost and fertilized with a balanced, water-soluble fertilizer (N, P, K) [72]. All plants were grown under natural light conditions from April to September 2019. Throughout the growth period, daytime temperatures ranged from 31–36°C and nighttime temperatures ranged from 20–24°C, along with average daytime relative humidity fluctuated between 29% and 43%, and nighttime relative humidity ranged from 47% and 65%. To counter the impact of extreme heat and high evaporation during the leaf formation phase, the site received regular irrigation of 3–4 hours. After leaf growth, irrigation occurred three times a week, each session lasting 4–5 hours. The plants were grown to maturity, at which point they were harvested. Specific details for each population are given in Table 3. Collection sites and various climatic zones of the studied populations, are shown in Fig. 7.

**Table 3:** Panel of 228 *Cannabis sativa* L. and geographical and ecological parameters of the cannabis populations studied

**Origin**	**Province**	**Region**	**Population Number**	**Population Code**	**Population Size**	**Elevation** **(m)**	**Longitude** **(E)**	**Latitude** **(N)**	**Annual Rainfall** **(mm)**	**Annual Avg. temp.** **(C)**
Iran	Zanjan	Abhar	P1	Abh-01	8	1543	49.22	36.28	301.31	12.39
Iran	West Azerbaijan	Urmiyeh	P4	Urm-01	7	1362	45.07	37.54	327.1	11.2
Iran	Qazvin	Qazvin	P10	Qzv-01	4	1315	49.86	36 47	311.85	13.91
Iran	Qazvin	Qazvin	P11	Qzv-02	13	1315	50.12	36.74	310.2	14.2
Iran	Hamadan	Samen	P12	Sam-01	7	1858	48.71	34.20	324.79	13.53
Iran	Hamadan	Samen	P13	Sam-02	3	1858	48.19	34.50	309.2	13.10
Iran	Khuzestan	Dezful	P15	Dez-01	4	144	48.42	32.38	389.40	24.56
Iran	Sistan & Baluchistan	Zahedan	P19	Zah-01	3	1352	60.86	29.49	73.58	19.30
Iran	Hamadan	Malayer	P20	Mal-01	6	1729	48.82	34.29	456.98	12.43
Iran	Kurdistan	Saghez	P23	Sqz-01	7	1480	46.26	36.24	439.37	11.20
Iran	Kurdistan	Sannandaj	P25	San-02	6	1464	46.97	35.33	437.9	14.3
Iran	Kerman	Sirjan	P27	Sir-01	10	1754	55.68	29.43	138.52	17.81
Iran	Hamadan	Nahavand	P32	Nhv-01	7	1666	48.25	34.15	385.29	14.72
Iran	Fars	Fars	P33	Frs-01	8	1390	53.71	27.38	312.3	16.86
Iran	Ardabil	Mugan plain	P35	Dshm-01	4	1339	47.87	39.66	398.43	9.12
Iran	Kerman	Kerman	P36	Krmn-01	4	1761	56.58	30.15	123.43	17.01
Iran	Kermanshah	Kermanshah	P37	Krsh-01	6	1389	47.03	34.19	402.63	15.51
Iran	Kurdistan	Baneh	P38	Ban-01	2	1503	45.53	35.59	660.88	14.26
Iran	Kermanshah	Gahvareh	P40	Gahv-01	5	1476	46.41	34.34	402.63	15.51
Iran	Markazi	Arak	P41	Ark-02	2	1722	49.68	34.09	326.6	13.7
Iran	Markazi	Mahalat	P42	Mahl-01	5	1746	50.44	33.90	280.9	14.3
Iran	South Khorasan	Boshrouyeh	P45	Bsh-01	6	881	57.43	34.03	79.98	21.07
Iran	Qom	Qom	P50	Qom-02	2	933	50.87	34.65	131.9	18.3
Iran	Esfahan	Radan	P51	Rad-01	6	1571	52.52	33.20	127.87	16.4
Iran	South Khorasan	Tabas	P52	Tab-01	9	981	56.92	33.59	79.98	21.07
Iran	Semnan	Shahroud	P53	Shah-01	8	1381	54.96	36.42	137.8	18.2
Iran	West Azerbaijan	Uromiyeh	P54	Urm-02	5	1328	45.30	37.39	327.1	11.2
Iran	East Azerbaijan	Tabriz	P55	Tabr-01	6	1345	46.14	38.70	272.2	12.1
Iran	Sistan & Baluchistan	Saravan	P56	Sarav-01	12	1352	62.33	27.36	73.58	19.30
Iran	Markazi	Khomein	P57	Khom-01	10	1798	50.07	33.63	296.32	13.6
Iran	Kurdistan	Sannandaj	P58	San-03	12	1464	47.52	35.78	437.9	14.3
Iran	Esfahan	Kashan	P61	Kash-02	6	949	51.44	33.98	136.5	19.7
Iran	Fars	Shiraz	P62	Shrz-03	7	1488	52.36	29.33	329.3	18.0
Iran	Fars	Shiraz	P63	Shrz-04	13	1488	52.36	29.33	329.3	18.0
Iran	North Khorasan	Bojnoord	P64	Boj-01	5	1112	57.18	37.29	255.1	13.2

### Phenotyping of the GWAS panel

For each population, randomly selected plants of the middle rows for each genotype were labeled, and the phenotypic variation of 13 traits was characterized. The number of plants employed per population varied based on availability, as highlighted in Table 3. Phenotypes assessed included inflorescence characteristics, flowering time, plant morphology, sex and biochemical trait analysis. Traits were (i) number of days that elapsed from germination to the initiation of flowering (DTF), (ii) the number of days from germination to appearance of approximately 50% flowering within a population (DT50), (iii) plant height (H; m) from the soil surface to the topmost terminal inflorescence before harvest, (iv) sex expression as a binary variable, i.e., male vs. female (M, F), (v) crown length, (CL; measurement of the length of the main stem from the soil surface to the lowest branch, cm), inflorescence features: (vi) inflorescence length (iL; measurement of the length of the main inflorescence in both male and female inflorescences, cm) as well as the (vii) number of lateral pistillate inflorescences (Li), (viii) internode length (intL, measurement of the length between two nodes, cm), (ix) number of nodes (nN), (x) footstalk diameter (FD) at the widest part of the base with digital calipers (cm), (xi) number of leaves (nL) and (xii) analysis of the ∆9- tetrahydrocannabinol (THC Q, %) and (xiii) cannabidiol (CBD Q, %) content of the plant material using HPLC. Air-dried pistillate inflorescences were collected prior to the seed development were analyzed for content (% dry weight (DW)) of the cannabinoid compounds ∆9-THC and CBD. Refer to our publication [5] for information on the preparation of samples and the HPLC analysis of cannabinoids. Some characteristics, including Li, nL, DT50 and CL, were only measured in female individuals. Furthermore, it is noteworthy that all plants included in this study exhibited dioecious characteristics, with either male or female flowers. As a result, we recorded the sex expression (male or female) of each individual in every population.

### DNA extraction, library preparation and genotyping of the GWAS panel

The GBS method was utilized to genotype the GWAS panel, following the protocol delineated by Elshire et al. [15]. At the juvenile stage before sexual differentiation, leaf tissue was collected from a labeled single plant of each population, and small segments of the tissue were placed into 2 ml vials and freeze-dried. High molecular weight DNA was isolated from approximately 25 mg of freeze-dried tissue following a modified cetyl trimethyl ammonium bromide (CTAB) protocol [73] which included a step for RNase treatment, to remove any potential RNA contamination, as RNA can inhibit the DNA sequencing library preparation (https://dnatech.genomecenter.ucdavis.edu/faqs/which-dna-isolation-protocols-do-you-recommend-for-illumina-sequencing/). DNA extracts were quantified using a Qubit^TM^ Fluorometer (ThermoFisher Scientific). Individual DNA samples were diluted to 10 ng/μl using 0.5 M Tris-EDTA (TE) buffer, pH 8.0. As the sex of the plants was unknown, to cover all allelic variation within populations and sexes, the genomic DNA was extracted from all available plants per population. 100 ng of each genomic DNA template (in a 10 μl volume) was used for library construction using a single digestion with restriction enzyme *ApekI* and ligated to unique 4–8 sequence barcode adapters. Five μl aliquots of adapter-ligated DNA samples were pooled in a single tube to produce 96-plex libraries. The pooled DNA was PCR-amplified using *Phusion® High-Fidelity PCR Kit* (NEB*®*), followed by purification with a *Monarch®* PCR & DNA Cleanup Kit (NEB*®*). Standard experimental conditions, as described by Elshire et al. [15], were followed for restriction, ligation, and PCR amplification. The purified DNA library was quantified and validated using a Bioanalyzer (Agilent Technologies). The 96-plex libraries were sequenced on a single lane of Illumina HiSeq™ 4000 platform as single-end 100 (SR100) base pair reads at the UC Davis Genome Center (Davis, CA, USA; https://genomecenter.ucdavis.edu/).

#### Demultiplexing, data quality control, and read filtering

The Stacks pipeline was used for GBS data analysis [74]. Demultiplexing and trimming the sequence reads were performed using *process_radtags* script, which trims adapter sequences and filters low-quality reads <50 bases. Samples with <100,000 reads were removed before analysis. To elucidate the genetic relationship among Iranian cannabis and marijuana and fibre type accessions, we integrated our data with two public datasets. This included marijuana data consisting of 81 samples and hemp data consisting of 43 samples originally prepared by Sawler et al. [17], and obtained from the NCBI SRA BioProject: PRJNA285813. Additionally, we incorporated 95 cannabis samples including 70 from Iran, 2 from Afghanistan and 26 accessions provided by CGN and IPK, as previously reported by Soorni et al. [28], and accessed from the BioProject: PRJNA419020.

#### Mapping, SNP variant calling and SNP filtering

We used a reference-based pipeline for sample alignment to generate consensus sequences. Trimmed sequence reads were aligned to the reference *C. sativa* 'CBDRx' assembly (cs10 v.1.0) as the most complete and contiguous chromosome-level assembly available at the time of analysis [8, 12], using the sequence alignment tool bowtie2 (http://bowtie-bio.sourceforge.net/bowtie2/manual.shtml#the-bowtie2-aligner) with the very-sensitive-local settings. This resulted in an average mapping rate of approximately 80%. The *ref_map.pl* script within the Stacks environment was utilized to call genetic variants (SNPs), and the "*populations"* program of the Stacks pipeline was used to filter the identified SNPs and estimate population genetics statistics like the fixation index (F_ST_) for genetic relationship analysis and Hardy-Weinberg equilibrium (hwe). The filtration criteria applied were as follows: requiring a locus to be present in a minimum of 10 populations for processing; setting a minimum of 50% individuals per population to process a locus for that population; necessitating a minimum of 50% individuals across populations for locus processing; and specifying a minimum minor allele frequency (MAF) of 0.05 for processing nucleotide sites at a locus. PLINK V 1.9 [75] was used for further filtering for both datasets (derived from this study and the publicly available data). Individuals with a genotyping call rate < 99%, SNPs with a genotyping call rate < 99%, and those exhibiting significant deviations from Hardy-Weinberg equilibrium (*P*-value <10^−6^) were excluded from the analysis [76]. Following this filtering process, the genetic data from both groups were combined, and only the SNPs that were common to both groups were selected. The set of obtained SNPs was used for subsequent analysis, including population structure analysis, heterozygosity and F_ST_ analysis and association analysis.

### Population structure analysis

*Admixture* 1.3.0 [77] was utilized to estimate the most likely number of clusters (K) into which the accessions could be grouped and their degree of admixture. The value of K that best fits the data was determined based on the lowest cross-validation (CV) error. Accessions were assigned to clusters based on the probabilities of belonging to one of the clusters derived from the matrix of contributions, Q. *Admixture* was run for each possible group number (K = 1 to 10). In addition, to visualize the genetic relationship and similarity among samples, a principal component analysis was carried out on a combined dataset of 431 samples. The analysis utilized ggplot2 (V3.4.4) for plotting [78], plotrix (V3.8.4) for zooming the plot, and tidyverse (V2.0.0) for eliminating duplicate samples [79] in R (V4.3.1). Following the quality control filtration process, this dataset consisted of 196 Iranian samples from the current study, 93 cannabis samples previously studied by Soorni et al. [28], as well as 47 individuals from a hemp population and 95 individuals from a marijuana population studied by Sawler et al. [17].

### Heterozygosity and F_ST_ analysis

Heterozygosity was estimated for each individual using PLINK V1.90 and then averaged within each group [75]. Additionally, we used R (V4.3.1) to generate plots.

Due to the limited number of individuals within some populations, we classified the studied Iranian populations into four larger groups based on their geographical distribution and associated climatic patterns. These groups include east and southeast, northeast, south and west and northwest populations. The F_ST_ value was calculated by fsthet package [80] in R (V4.3.1) for each pair of populations to measure genetic differentiation among populations. F_ST_ analysis was conducted three times: firstly, among the four geographic populations of Iranian samples; secondly, using combined data from this study and two public datasets containing previously studied Iranian samples, as well as other marijuana and hemp populations (NCBI SRA BioProject: PRJNA419020 and PRJNA285813), and in the third analysis, all Iranian populations from this study were treated as a single population and combined with two above-named public collections. The F_ST_ plots were created in R (V4.3.1) using the qqman package [81] to visualize the relationships between populations based on the F_ST_ values. We then conducted a thorough analysis of significant SNPs for each pair to identify the specific SNP markers contributing to the observed differences.

### IBD Test

We employed PLINK V1.90 software to conduct pairwise IBD analysis, investigating first-degree and second-degree relationships among individuals by assessing the proportion of SNPs where zero, one, or two shared IBD alleles were present, represented by Z0, Z1, and Z2, respectively. Subsequently, relatedness was quantified using the PI_HAT parameter, indicating the proportion of SNPs in IBD between individual pairs [51].

### Association analysis

To identify the associations between genetic varaints and trait performance, GWAS was carried out to estimate SNP effects. Studies indicates that employing a linear mixed model that incorporates population and family structures is currently the most effective approach for mitigating the impact of population stratification [82]. The statistical model used for GWAS analysis is based on the mixed linear model as follows:

$$y=Xb+Z\gamma +Mu+e$$

Where y is a vector representing the phenotype, b is a vector representing fixed effects (group), γ is a vector representing fixed effects of markers, and u is a vector representing random effects (e.g., PC1, PC2, and PI_HAT). X, Z, and M are matrices relating observations to the effects of fixed factors, fixed SNP effects, and random genetic effects, respectively, and e is a vector representing random residuals with e ~ N(0, I σe2).

The analysis initially utilized a mixed linear model through GCTA V1.94.1 software [83]. Additionally, for undertaking GWAS for sex as a qualititative trait, we employed PLINK V1.90 with case and control anaylsis. Subsequently, we applied Bonferroni testing using PLINK's --assoc, --perm, and --adjust functions to effectively control for potential false positives. Finally, Manhattan plots and the Q-Q plots were constructed in R (V4.3.1) using the qqman package [81] to visualize the genome-wide association signals.

Furthermore, GCTA V1.94.1 software was utilized to measure the SNP-based heritibility (h^2^) for each trait [84]. The variance of total additive genetic effects is defined as ${\sigma }_{g}^{2}$= p . ${\sigma }_{\beta }^{2}$. The GCTA software was used to estimate the variance components ${\sigma }_{g}^{2}$ and ${\sigma }_{e}^{2}$. The SNP heritability is estimated as follows:

$${h}_{g}^{2}=\frac{{\sigma }_{g}^{2}}{{\sigma }_{g}^{2}+{\sigma }_{e}^{2}}$$

### Functional annotation of candidate SNPs

In addition, functional annotation of the candidate SNPs identified in the GWAS analysis was performed. For each trait, the significant markers were compared and annotated using the annotated reference genome (cs10, GCF_900626175.1, https://www.ncbi.nlm.nih.gov/genome/annotation_euk/Cannabis_sativa/100/) and the annotated genes were identified using the NCBI Genome Data Viewer (https://www.ncbi.nlm.nih.gov/genome/gdv/browser/genome/?id=GCF_900626175.2) [9].

## Supplementary Information

Supplementary Material 1.

## Data Availability

Our Sequence Read Archive (SRA) records will be accessible with the following link after the indicated release date: https://www.ncbi.nlm.nih.gov/sra/PRJNA1076947. Accession to cite for these SRA data: PRJNA1076947. Temporary Submission ID: SUB14220748. Release date: 2024-12-01.

## Abbreviations

THC: Tetrahydrocannabinol
CBD: Cannabidiol
GBS: Genotyping-By-Sequecning
SNP: Single Nuclotide Polymprphism
DTF: Days To First flowering
DT50: Days to 50% flowering
H: Height
M, F: Male, Female
CL: Crown Length
iL: inflorescence Length
Li: number of inflorescences
intL: internode Length
nN: number of Nodes
FD: Footstalk Diameter
nL: number of Leaves
THC Q: ∆9- Tetrahydrocannabinol content
CBD Q: Cannabidiol content
