# Supplementary material for: Population genomics of a natural Cannabis sativa L. collection from Iran identifies novel genetic loci for flowering time, morphology, sex and chemotyping
Source: BMC Plant Biol. 2025 Jan 21;25:80. doi: 10.1186/s12870-025-06045-4 (PMC11748290; doi:10.1186/s12870-025-06045-4)
Supplement: Supplementary file 1 — Supplementary Material 1. [file 12870_2025_6045_MOESM1_ESM.zip › Supplementary/Supplementary Figures.docx]

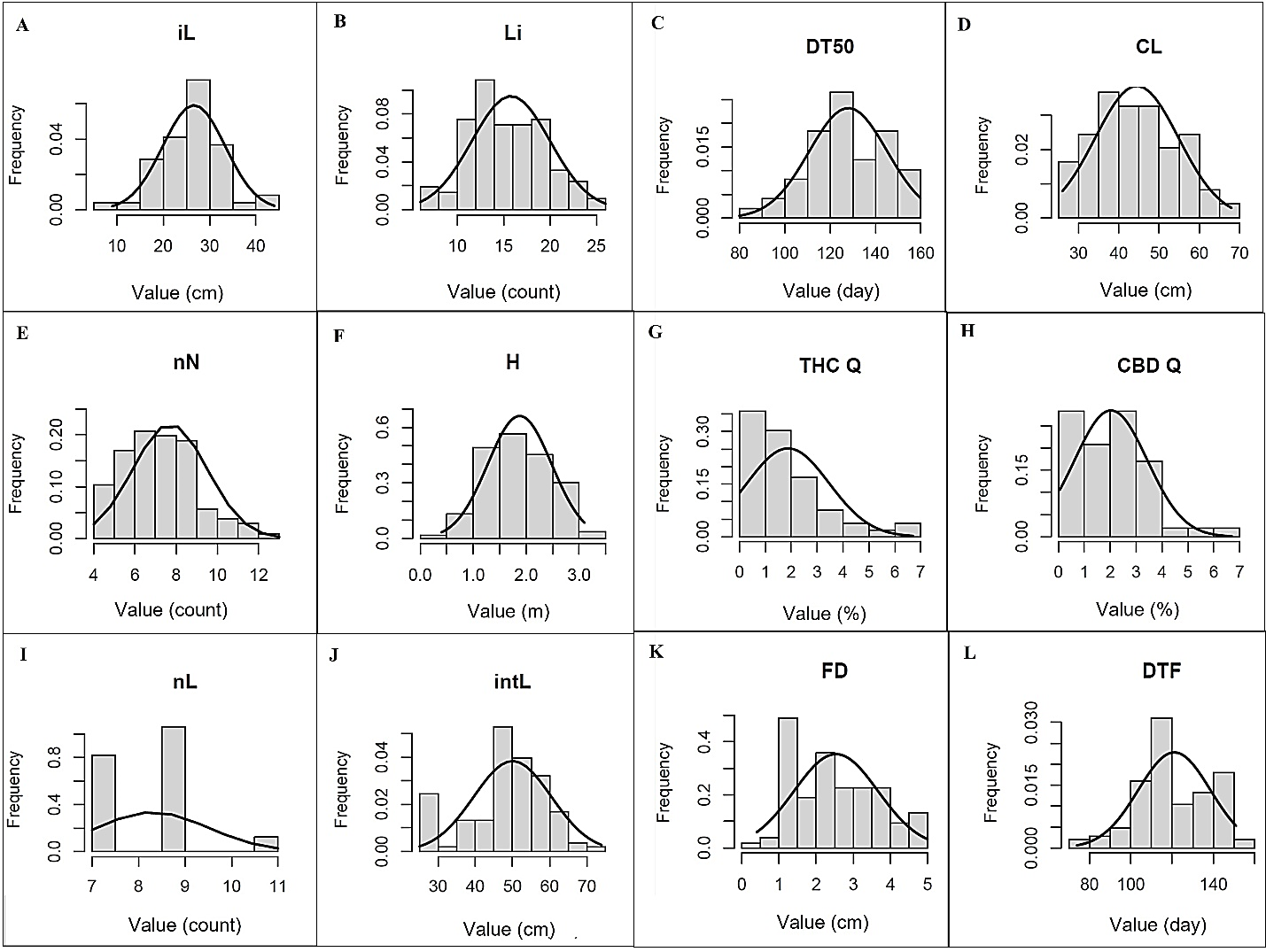


**Supplementary Figure 1.** Frequency distribution for investigated traits of cannabis genotypes used in this study. (**A**) inflorescence length (iL), (**B**) number of lateral pistillate inflorescences (Li), (**C**) number of days to 50% flowering (DT50), (**D**) crown length (CL), (**E**) number of nodes (nN), (**F**) plant height (H), (**G**) ∆9- tetrahydrocannabinol quantity (THC Q) (**H**) cannabidiol quantity (CBD Q), (**I**) number of leaves (nL), (**J**) internode length (intL), (**K**) footstalk diameter (FD), and (**L**) number of days to the initiation of flowering (DTF). The diagrams were generated using R (V4.3.1).


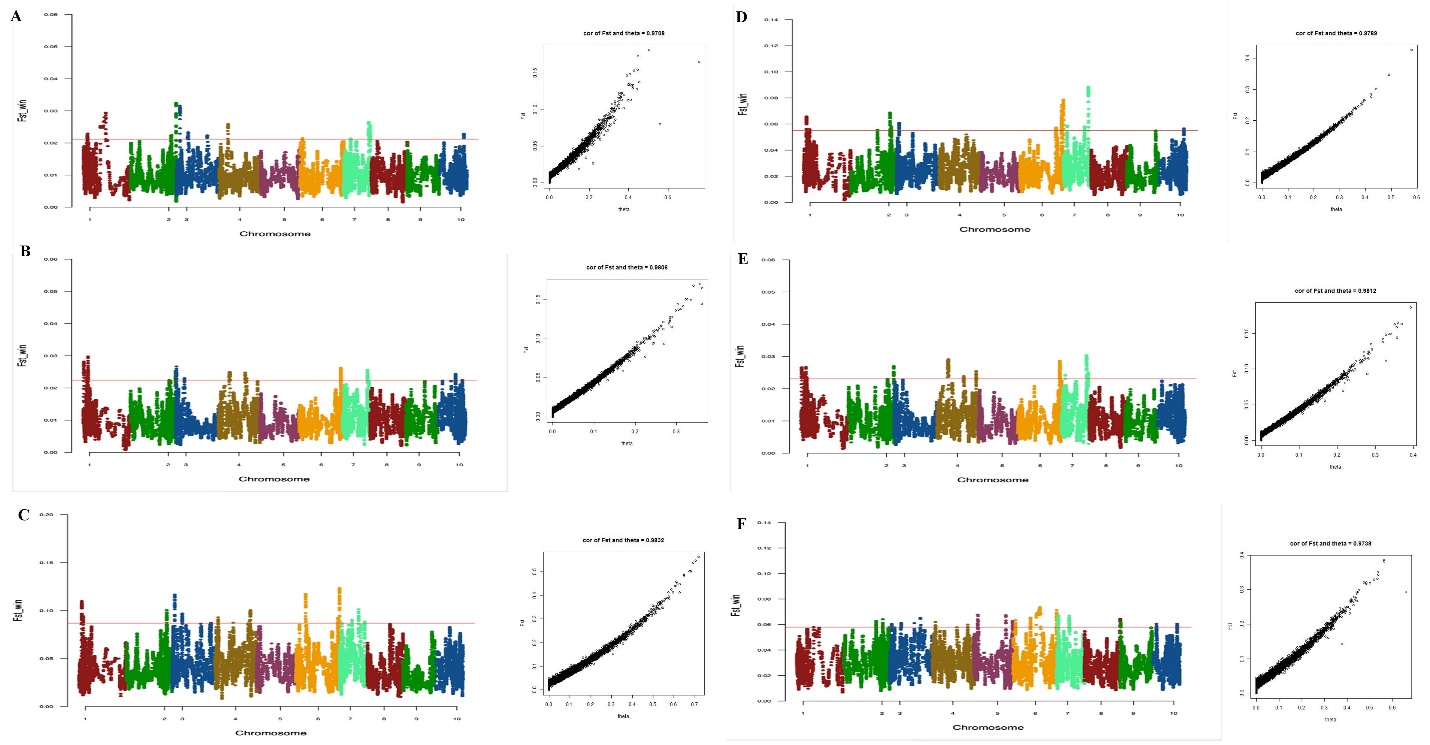


**Supplementary Figure 2.** Pairwise F_ST_ Manhattan plots for studied Iranian populations classified based on geographical distribution and climatic variables: (**A**) northeast: west and northwest (**B**) south: west and northwest (**C**) east and southeast: northeast (**D**) east and southeast: south (**E**) east and southeast: west and northwest (**F**) northeast: south.


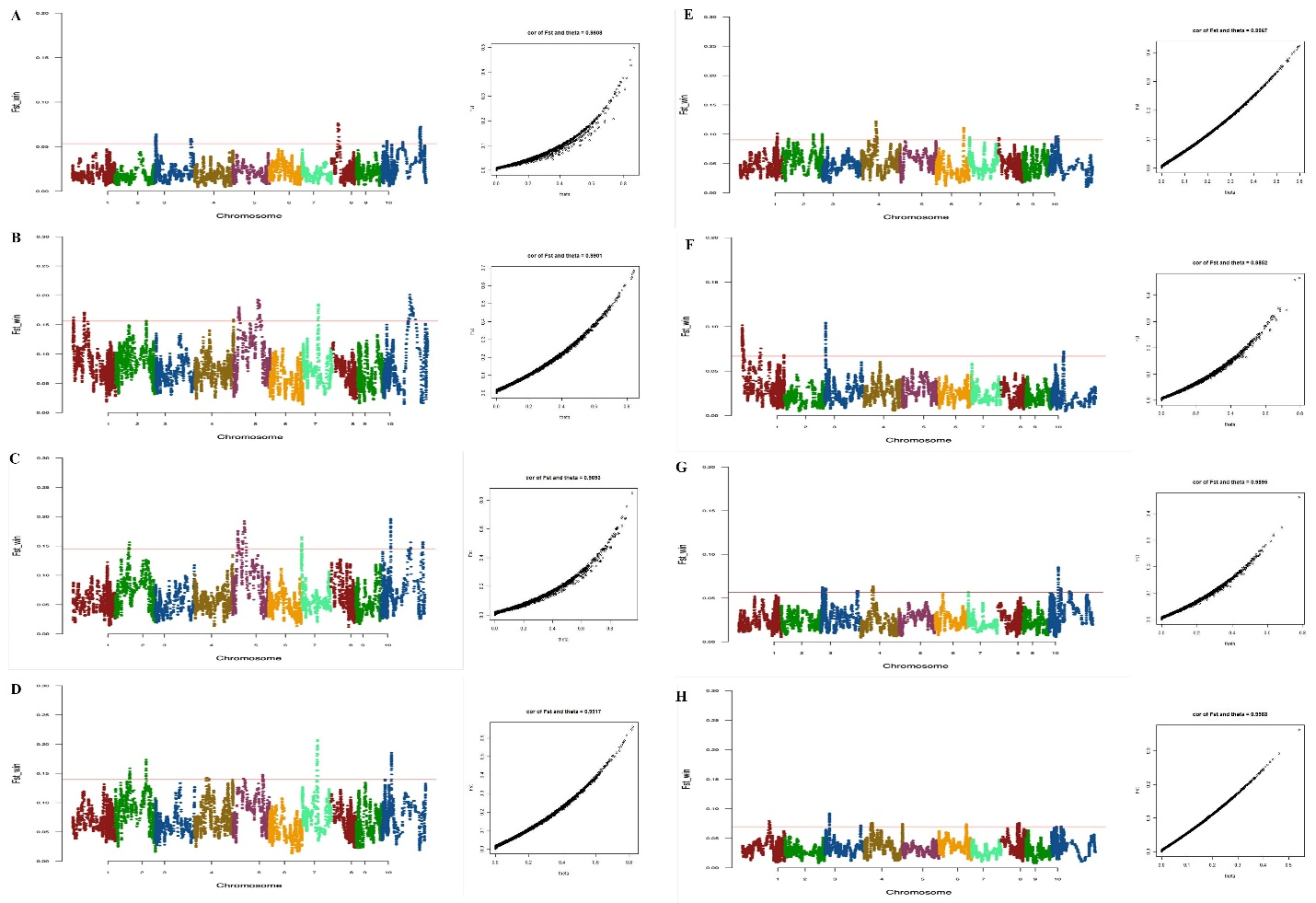
**Supplementary Figure 3.** Pairwise F_ST_ Manhattan plots for four geography-based Iranian populations and previously studied Iranian samples and global collections including hemp and marijuana. (**A**) marijuana population: northeast (**B**) hemp population: east and southeast (**C**) hemp population: northeast (**D**) hemp population: south (**E**) hemp population: west and northwest (**F**) marijuana population: east and southeast (**G**) marijuana population: south (**H**) marijuana population: west and northwest.


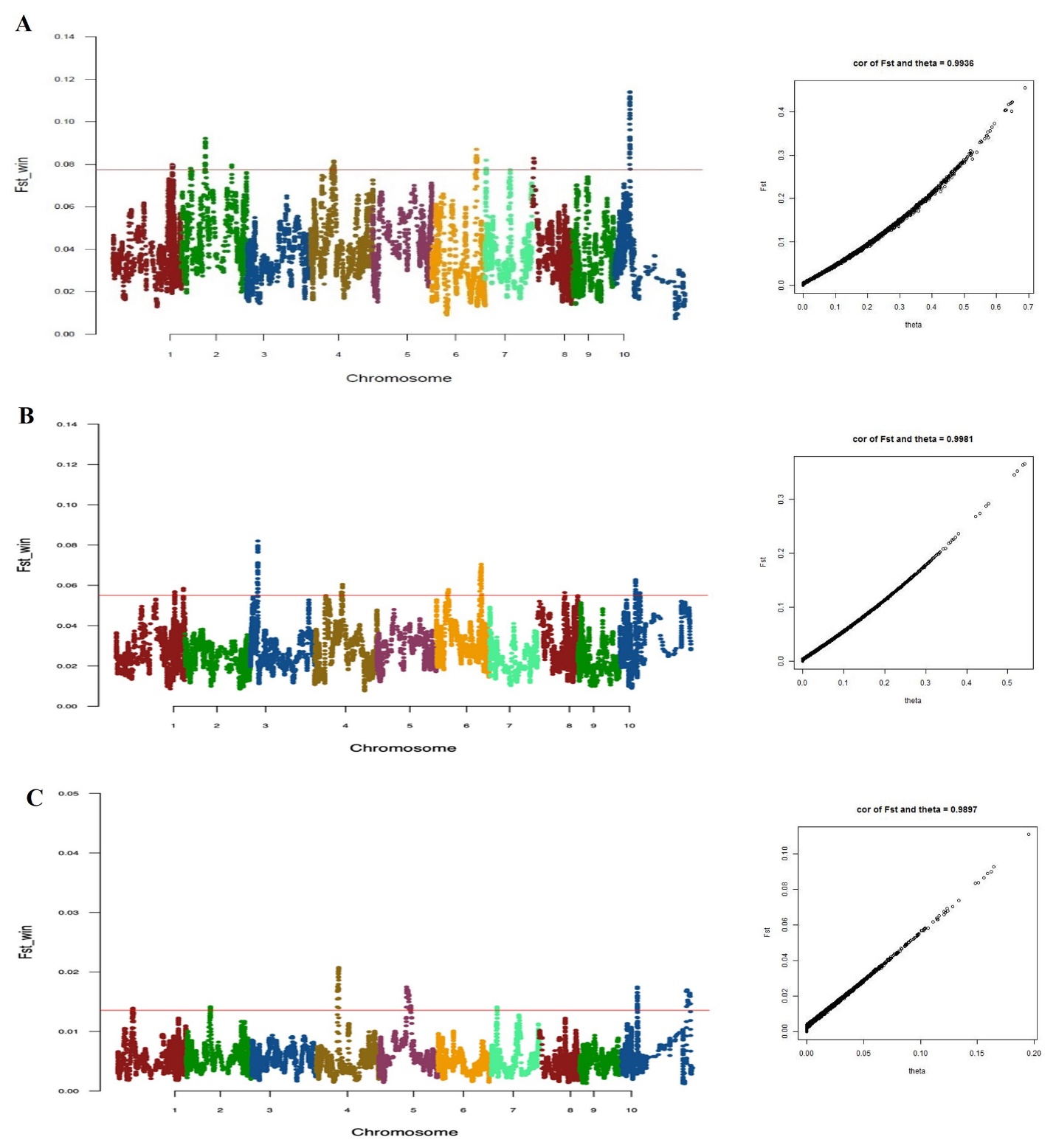


**Supplementary Figure 4.** Pairwise F_ST_ Manhattan plots for Iranian samples of this study as a single population and hemp/marijuana and previously studied Iranian samples. (**A**) Iranian samples of current study: hemp population (**B**) Iranian samples of current study: marijuana population (**C**) Iranian samples of current study: previously studied Iranian samples.
